# Supported Ionic Liquid Phase Catalysts Dedicated for Continuous Flow Synthesis

**DOI:** 10.3390/ma16052106

**Published:** 2023-03-05

**Authors:** Piotr Latos, Anna Wolny, Anna Chrobok

**Affiliations:** Department of Organic Chemical Technology and Petrochemistry, Faculty of Chemistry, Silesian University of Technology, 44-100 Gliwice, Poland

**Keywords:** ionic liquid, flow synthesis, heterogeneous catalysts

## Abstract

Heterogeneous catalysis, although known for over a century, is constantly improved and plays a key role in solving the present problems in chemical technology. Thanks to the development of modern materials engineering, solid supports for catalytic phases having a highly developed surface are available. Recently, continuous-flow synthesis started to be a key technology in the synthesis of high added value chemicals. These processes are more efficient, sustainable, safer and cheaper to operate. The most promising is the use of heterogeneous catalyst with column-type fixed-bed reactors. The advantages of the use of heterogeneous catalyst in continuous flow reactors are the physical separation of product and catalyst, as well as the reduction in inactivation and loss of the catalyst. However, the state-of-the-art use of heterogeneous catalysts in flow systems compared to homogenous ones remains still open. The lifetime of heterogeneous catalysts remains a significant hurdle to realise sustainable flow synthesis. The goal of this review article was to present a state of knowledge concerning the application of Supported Ionic Liquid Phase (SILP) catalysts dedicated for continuous flow synthesis.

## 1. Introduction

In times of increased environmental awareness, challenges of creating sustainable protocols for many organic reactions are becoming increasingly important. The most important issues for the selection of an ideal catalyst are high activity, selectivity and stability. The catalyst should also be environmentally benign [1].

Many important chemical reactions take place in the presence of catalysts. Among them, acidic catalysts are the most abundant in quantity. Refinery alkylation and alkane isomerisation are typical examples. Conventional methods for these processes often require strong acids, such as HF or BF_3_, which brings some restrictions. They are very often hazardous, volatile, corrosive, toxic, and used in excess of the raw materials. Strong acids produce large amounts of wastes, causing environmental problems. Furthermore, limited solubility of the substrates requires additional solvents. Nano-dimension polyoxometalates with high Brønsted acidity and high thermal stability can also be an alternative to acids and are also widely used in catalysis [2]. These facts have caused huge interest in exploring alternative catalysts, which can overcome the limitations described above [3].

A great alternative for conventional catalysts are task-specific ionic liquids (ILs), a range of non-volatile liquids, comprising an organic cation and an organic or inorganic anion. ILs can dissolve both lipophilic and polar compounds, and are widely reported to be active homogeneous catalysts. The main drawbacks in their application are high cost and difficulties with purification after the synthesis [4]. On the other hand, two aspects of the use of ILs in catalysis are crucial: the solubility of the reaction components (raw materials, products and intermediate forms or additional substances and catalysts) and specific interactions that may occur between the ionic liquid and the components present in the reaction system. High solubility of organic and inorganic compounds resulting from a high dipolar, dispersive or electronic force will allow for faster diffusion of reactants and increase the reaction rate, and consequently increase conversion of regents. Occurrence of specific interactions may lead to the formation of a characteristic intermediate product, which will allow for obtaining higher selectivity [5].

While homogeneous catalysis generally offers very high activity and homogeneous distribution of the active sites, it is not an ideal solution, especially with regards to separation and reuse. The solid catalyst can either be used in a fixed bed configuration or simply filtered off or centrifuged from a stirred tank reaction and then, in many cases, reactivated for reuse, higher reaction rate and selectivity. The disadvantages of heterogeneous catalysis include added synthesis costs, possible blocking of catalyst sites and often limited activity. Mechanisms of reactions in the presence of heterogeneous catalysts are very often complicated and consists of several steps: 1. Diffusion of reagents through the layers surrounding the catalyst particle, 2. Intramolecular diffusion of reagents through the pores of the catalyst to active sites, 3. Adsorption of reagents in active sites, 4. Reaction involving the formation or transformation of adsorbed intermediates, 5. Desorption of products from active sites, 6. Intramolecular diffusion of products through the pores of the catalyst, 7. Diffusion of products through the layers surrounding the catalyst particle. However, heterogenization of the catalysts is one of the most widely favoured green chemical technologies. The simplicity of catalyst separation and the possibility of using a fixed-bed reactor make this idea attractive. The possibility to construct a well-defined active site, test its catalytic performance, and assess a structure-activity relationship is necessary for the design of active heterogeneous catalyst [6]. This knowledge is still limited, in particular for flow systems.

A big group of heterogeneous catalysts are based on nanoparticle structure, which enables faster conversion of reagents, higher yields of products and simple post-process regeneration and recovery [7]. Nanoparticles are particles with a size of 1–100 nm, which can be synthesised using the following methods, i.e., sol-gel [8], hydrothermal [9], sol-gel auto-combustion [10], microemulsion [11], chemical coprecipitation [12], spray pyrolysis [13], etc. Due to the developed contact surface and the appropriate structure and shape of the molecules, nanoparticles can also affect the reaction mechanism, thanks to which higher selectivity can be obtained. Nanocatalysis has recently gained interest in both scientific research and industry, due to the fact that it combines the advantages of homogeneous and heterogeneous catalysis while eliminating their disadvantages. 

The use of ILs in homogenous system generates many costs. High viscosity also makes it difficult to achieve high performance. The separation of ILs after a large-scale process is another serious problem. Therefore, many studies have been devoted to immobilizing ILs to improve catalyst separation and reduce costs by using heterogeneous ILs [14,15,16,17,18]. Additionally, their use in a continuous system with a fixed-bed catalyst can increase catalytic efficiency and assess their belonging to industrial processes.

**Figure 1:**
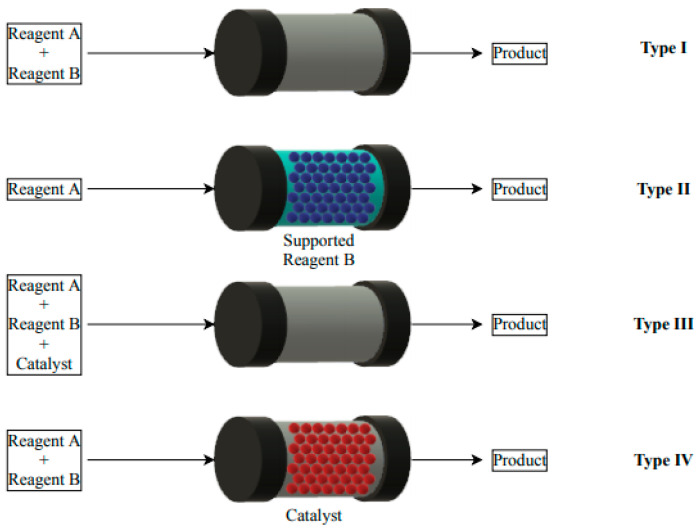
Examples of flow reactions in organic synthesis.

Continuous flow systems can be divided into four categories according to the type of reaction (Figure 1) [19,20]. The simplest case is a situation where two substrates are added to the reactor (Type I), and the product is constantly collected at the reactor outlet. This method can be used for non-catalytic processes that use the possibility of constant product collection. Unfortunately, in this case, the product is contaminated with unreacted substrates and it must be purified. The second type involves packing the substrate on the carrier inside the reactor and the substrate stream is passed through the reactor (Type II). The disadvantage of this solution is the need to constantly replace the substrate inside the reactor. Another category of flow systems is an example of homogeneous catalysis. Two substrate streams are introduced into the reactor, where the catalyst is dissolved (Type III). It is a simple approach to catalytic reactions at flow conditions. Unfortunately, in this case, the product must be further purified from the catalyst. This process is difficult to carry in a continuous system for a longer time, due to the need of removing the catalyst. The last type of flow system is the use of a heterogeneous catalyst in the reactor space, through which the substrate streams are fed in a continuous system flow (Type IV). The effect of using such a system is that there is no loss of catalyst, and the product is obtained with high purity. From a process point of view, this catalytic system is ideal with the possibility of avoiding continuous catalyst release and the product is only contaminated with unreacted substrates or by-products. These systems are most often used in continuous flow [21,22,23,24,25]. Packed-bed reactors are among the most used, due to easy assembly and high possibility of catalyst loading. Nevertheless, heat and mass transport limitations can easily occur, and significant pressure drop can arise. In order to develop flow systems with column-type fixed-bed reactor (update form batch rector) the key parameters should be determined with addition to the yield and selectivity: productivity per time, productivity per catalyst, space-time yield, TON (Turnover Number), and TOF (Turnover Frequency) under a particular space velocity.

The synthesis of chemicals in a continuous system is a key technology. It allows us to run processes in a shorter reaction time and receive products with high yields and selectivity. The reactions can be scaled up and optimised in a very simple way. The reactor design allows for high safety processes undergone at elevated pressure. The use of a continuous system allows the better control of the life and stability of the catalyst. Investigating parameters, such as productivity per time, productivity per catalyst, space-time yield, TON, and TOF under a particular space velocity will allow for a better understanding of the flow system and may be more important than the selectivity and yields for a batch process. Nowadays, a great challenge is to develop a process that limits the amount of by-products formed while maintaining high conversion. To achieve these goals, more efficient heterogeneous catalysts should be developed that would allow high atom economy to be achieved under continuous flow conditions [26,27].

To summarise, SILP (Supported Ionic Liquid Phase) catalysis in continuous flow systems is in the development phase, opening new possibilities of application, especially in the chemical industry, for the production of fine chemicals.

## 2. SILP Catalysts in Continuous Flow Reactors

**Figure 2:**
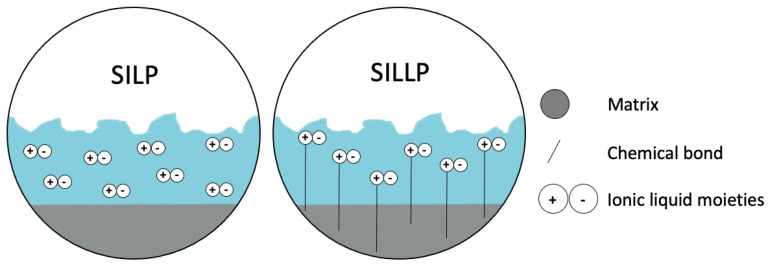
Illustration method of depositing the ionic liquid on the support, SILPs (SILP: IL film disp–ersed on the surface of support and SILLP: IL bound by chemical bonds). Reprinted with permission from Ref. [31]. Copyright 2022, MDPI.

The concept of SILP catalysis involves the use of a thin film of IL adsorbed or bonded covalently onto the solid surface [28]. The ILs can be bound to a surface either via covalent bonds, e.g., between silanol groups and the anion or the cation of IL, or by the Van der Waals forces (Figure 2) [29]. SILP catalysis is a method garnering considerable interest for many chemical processes due to its many advantages over conventional biphasic or homogeneous systems. The recovery and reusability of catalyst in supported ionic liquid phases (SILP) are generally considered to be major advantages of these systems. The ionic liquid support materials can enhance stability of the catalyst and prevent its leaching into the reaction mixture, enabling high catalyst activity and efficiency over multiple reaction cycles. The ionic liquid supports can also make the catalyst easier to separate and recover after the reaction is complete, allowing it to be reused in subsequent reactions. This improvement of the stability and recoverability of catalyst in SILP systems can help to reduce the cost and environmental impact of chemical processes, as well as improve their overall efficiency and performance. However, as with any catalytic system, there can be challenges and limitations associated with the recovery and reuse of catalyst in SILP systems, such as catalyst degradation, leaching, and deactivation. These issues can be addressed through the development of more robust and stable catalytic systems, as well as improved methods for catalyst separation, recovery, and reuse [30]. The activities of these systems are the same as, or slightly higher than, conventional biphasic catalysis, and the catalysts demonstrate good stability with no leaching of the active species or the IL. IL can play different roles in SILP processes. First IL can be a catalyst itself (task-specific ILs) or can act as the support for the immobilization of the corresponding catalyst (organometallic catalyst, metal nanoparticles, organocatalysts or enzyme) [28]. Supported acidic ILs catalysts can be characterised by improved activity and selectivity compared with the free ILs. Compared with other well-known acidic catalysts, e.g., silica, alumina or zeolites. Immobilised ILs also have additional advantages. The most important advantage is the easily tuneable acidity and the possibilities given by the choice of support material and its properties. However, properties such as the surface area and pore width cannot be easily tuned. The specific surface of the support and its porosity mainly depends on the synthesis method, i.e., temperature, components addition, form, pressure, etc. Furthermore, by changing the length of the side alkyl chains of the cation, the hydrophilicity or hydrophobicity of the surface can be enhanced. In the literature, processes with solid catalysts consisting of immobilised ILs could be performed either in a batch process or by continuous flow operation.

The most popular method for SILP application is the batch system [17,28,32,33,34,35,36,37,38,39]. Numerous examples concerning the use of various carriers, such as silica, polymer, carbon nanotubes, cellulose, Merrifield resin, aluminium, Ti oxoclusters, titanium and zirconium oxide for the broad range ILs were described. These systems were used for broad ranges of chemical reactions, e.g., applied to improve the performance of homogeneous transition-metal catalysts for hydroformylation, metathesis reactions, carbonylation, hydrogenation, hydroamination, coupling reactions and asymmetric reactions [28]. Homogeneous catalysis and SILP catalysis are two approaches to chemical production and processing that have different advantages and disadvantages. Homogeneous catalysis involves using a soluble catalyst in the same liquid phase as the reactants. The catalyst is dispersed throughout the reaction mixture, and the reactants diffuse to the catalyst for the reaction to take place. On the other hand, SILP catalysis involves using a solid support to hold the catalyst, and the reactants come into contact with the supported catalyst, initialising the reaction.

Ones of the key advantages of SILP catalysis over homogeneous catalysis is the improved catalyst recovery and reusability. In SILP systems, the catalyst can be easily separated and reused, reducing the environmental impact and improving the economic aspects of the whole chemical process. Additionally, SILP systems have been shown to provide improved selectivity for specific reactions, leading to higher yields and greater purity of the final product. The stability of SILP systems is also higher compared to traditional homogeneous catalysts, which leads to longer reaction times and greater efficiency. The use of SILP systems also provides greater control over reaction conditions, such as temperature, pressure, and flow rate, leading to improved reaction performance and efficiency. Furthermore, the use of SILP systems can help to reduce the amount of solvent required for chemical processes, leading to a more environmentally friendly approach.

In conclusion, while both homogeneous and SILP catalysis have their own advantages and disadvantages, SILP systems offer several key benefits over homogeneous catalysis, making it an attractive alternative in many cases. These benefits include improved catalyst recovery and reusability, enhanced selectivity, increased stability, improved control, and reduced the solvent usage [32]. 

In this paper, the author discusses the advancements in SILP materials and their applications in a flow system reactor. Previously, Mehnert [40] discussed the initial use of SILPs in catalysis. Subsequently, Sokolova et al. [41] conducted a review of the flow processes that utilised catalysts immobilised on monolithic SILLPs (Supported Ionic Liquid-Like Phases). Later, Skoda-Földes [42] summarised the utilization of supported AILs (Acidic ionic liquids) in organic synthesis. Furthermore, Hartmann et al. [43] analysed inorganic materials used for SILLP synthesis and briefly explained their role in catalysis. Subsequently, Amarasekara [44] analysed the characteristics of AILs and described the use of acidic ionic liquids as SILP/SILLP. Gruttadauria et al. outlined covalently-supported ionic liquid phases (SILLP) as both matrices and catalysts [45], while Alinezhad et al. emphasised the role of BAILs as SILLP in organic catalysis [46]. Later, Swadźba-Kwaśny et al. [47] briefly discussed the use of Lewis ILs immobilised into a solid matrix. Leitner et al. [48] described the applications of SILP and SILLP based on nanoparticles in organic catalysis. Furthermore, Vekariya [49] briefly mentioned SILPs in a review of ILs in organic transformations. Later, Haumann et al. [50] presented 15 years of experience using SILP/SILLP catalysts in hydroformylation reactions, both in liquid and gas phases. Additionally, Freire et al. [51] described the techniques for immobilizing ionic liquids, the different types of materials used, and their applications. Maciejewski et al. discussed the role of ionic liquids (ILs) in heterogeneous catalysis, including the use of supported IL phase catalysts (SILPC), solid catalysts with ionic liquids (SCILL), and supported ionic liquid catalysis (SILC) techniques, as well as porous ionic liquids [52]. Additionally, Lozano et al. outlined the use of SILP and SILLP as a matrix for the immobilization of enzymes in organic synthesis [53]. Chrobok et al. also discussed SILP/SILLP biocatalysts that are based on nanoparticles and their applications in biocatalysis [54]. In our previous work, catalytic applications of the silica-based SILLP materials in the organic synthesis were presented (Chrobok et al.) [31]. The goal of this study is to fill the time gap and gather information on the use of SILP catalysts in flow reactor systems for organic synthesis.

**Figure 3:**
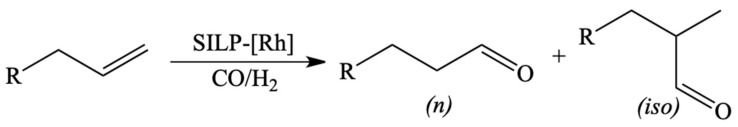
The synthesis of linear (n) and branched (iso) aldehydes in hydroformylation of olefines. Reprinted with permission from Ref. [56]. Copyright 2003, Springer.

SILP catalysis and continuous flow processes is a good marriage to design sustainable processes [26]. This issue is very attractive for both cases when reactants are in a gas or liquid phase. In both cases, IL is mostly just a carrier for metal catalyst. Historically, the SILP catalysis in flow systems started to be studied first in the gas phase, and up to now is the most popular and best-described technique. Generally, only silica, a few examples of Al_2_O_3_, and carbon nanofibers were used as supports for ILs. [49,55,56,57,58,59,60,61,62,63,64,65,66,67,68,69,70,71,72,73,74,75,76] Two chemical deposition techniques are mainly used for silica. The first one concerns immobilization via a suitably modified cation with alkoxysilyl groups. The second method assumes immobilization via an anion [77]. In the IL layer, deposited on a silica support, Ru complexes were dissolved and used for hydroformylation of olefins (Figure 3) [49,55,56,57]. The modification of the surface of silica with IL allowed for improving the solubility of reactants in the IL layer, and increasing the conversion of reactants and improving selectivity in relation to obtaining linear products. 

**Figure 4:**
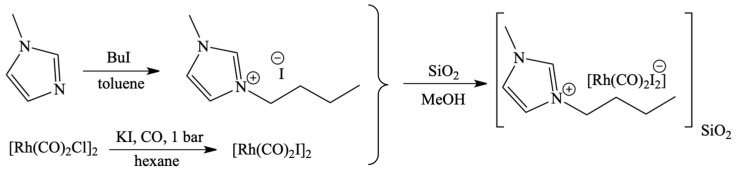
The preparation of [BMIM][Rh(CO)_2_I_2_]-[BMIM]I catalyst and immobilization on SiO_2_ surface. Reprinted with permission from Ref. [58]. Copyright 2008, Royal Society of Chemistry.

A similar catalytic system was used for the first time for the methanol carbonylation reaction, where the IL deposited on a high porosity silica carrier together with a rhodium catalyst was used in the gas phase (Figure 4) [58]. The use of SILP catalyst allowed for shortening the reaction time and caused a reduction in reactor sizes in the described technology.

**Figure 5:**
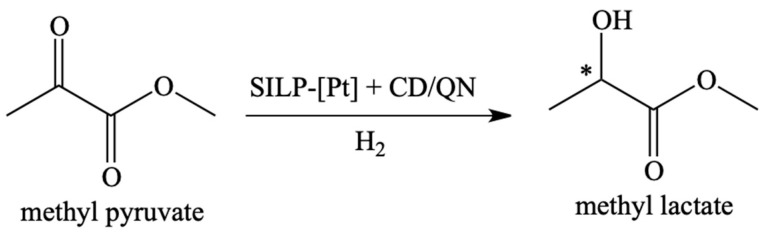
Application of SILP catalysts in the gas-phase asymmetric hydrogenation of α-keto esters. * Chiral atom. Reprinted with permission from Ref. [59]. Copyright 2013, Elsevier.

Rhodium catalyst was also used in the process of asymmetric hydrogenation of methyl pyruvate in the continuous gas phase using SILP catalysis (Figure 5) [59]. The most active catalytic systems were based on 1-ethyl-3-methylimidazolium ILs, such as bis(trifluoromethylsulfonyl)imide [EMIM][NTf_2_] and 3-hydroxypropyl pyridinium bis(trifluoromethylsulfonyl)imide [PrOHPyr][NTf_2_] supported on silica and a dissolved catalyst based on a ruthenium complex. Ester can be obtained with 80–84% yield for more than 50 h on stream. The use of IL allowed f increasing the stability of the catalyst. Moreover, the addition of the acidic IL [BMIM][HSO_4_] influenced the 50% increase of the enantioselectivity.

**Figure 6:**
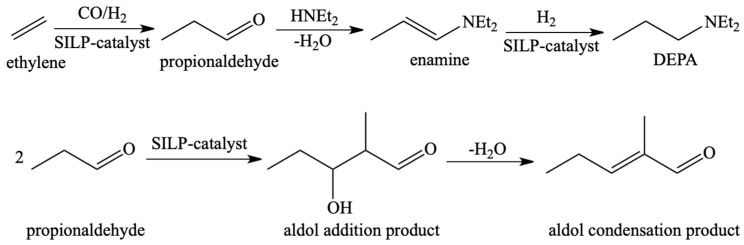
Reaction of hydroaminomethylation of ethylene with diethylamine. Reprinted with permission from Ref. [60]. Copyright 2013, John Wiley & Sons.

**Figure 7:**
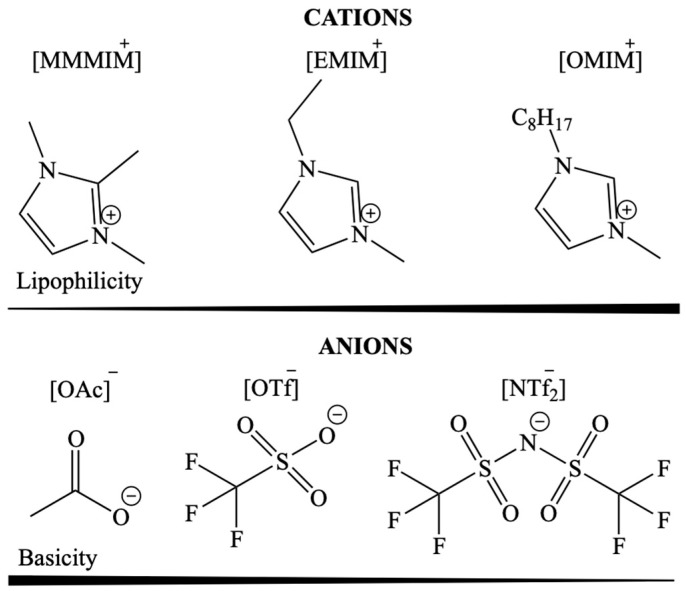
Lipophilicity and basicity nature of the cations and anions. Reprinted with permission from Ref. [60]. Copyright 2013, John Wiley & Sons.

Another example describes the use of a rhodium catalyst dissolved in IL on a polymer-based spherical activated carbon support in a gas-phase hydroaminomethylation (Figure 6) [60]. During the process, two parallel reactions occurred that allowed for the selective preparation of linear aldol condensation products. The resulting amine stabilised the intermediate product, reducing the amount of branched products. The ILs deposited on the support were characterised by various polarity and basicity (Figure 7). The highest TOF value (549 h^−1^) was obtained for the use of IL with the highest lipophilicity and the lowest basicity (1-methyl-3-octylimidazolium bis(trifluoromethanesulfonyl)imide) [OMIM][NTf_2_].

**Figure 8:**
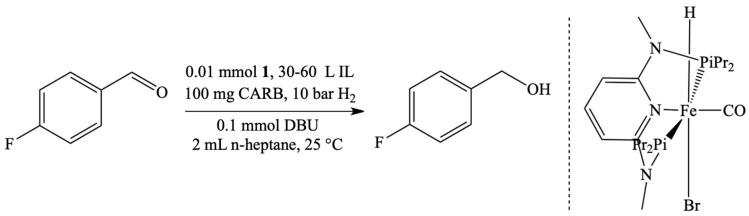
Application of SILP catalysts in the hydrogenation of 4-fluorobenzaldehyde. Reprinted with permission from Ref. [64]. Copyright 2018, Royal Society of Chemistry.

The continuous system in a gas phase was also used to obtain synthesis gas. IL (1-butyl-2,3-dimethylimidazolium chloride, [BMMIM]Cl or trifluoromethanesulfonate [BMMIM][OTf]), together with the ruthenium catalyst were deposited on γ-alumina or aluminium oxide hydroxide [61]. The most active system was based on [BMMIM]Cl, in which the additive of [{Ru(CO)_3_Cl_2_}_2_] was used. As a result, a high conversion of the raw material (71.2%) was obtained at 150 °C, comparing to commercial catalyst (47.5% at 180 °C). Another approach to hydrogenation involves dissolving of a rhodium catalyst in [EMIM][NTf_2_] layer and depositing it on silica [62,63] or polymer-based spherical activated carbon (Figure 8) [64]. The use of IL effected in increasing the conversion of reagents to 99%. 

**Figure 9:**
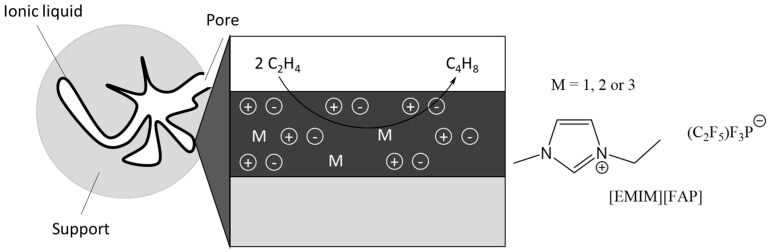
Illustration of Ni−based SILP catalyst system. Reprinted with permission from Ref. [65]. Copyright 2014, Royal Society of Chemistry.

**Figure 10:**
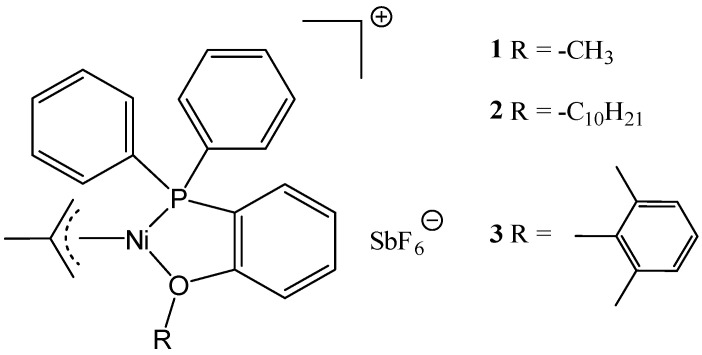
Nickel catalyst complexes applied in dimerization of ethene in the gas phase. Reprinted with permission from Ref. [65]. Copyright 2014, Royal Society of Chemistry.

Examples of application of other gas-phase SILP catalysts are less common, e.g., in dimerization of ethene in the gas phase with Ni complex catalyst dissolved in IL and deposited on silica (Figure 9 and Figure 10) [65]. The use of IL had a positive effect on the stability of the catalyst. In addition, the IL reduces the deactivation behaviour of the metal catalyst in the reaction mixture.

Gas-phase hydrogenation was also carried out in a continuous flow reactor with SILP catalyst. Palladium nanoparticles trapped in [BMIM][PF_6_] or [BMIMOH][NTf_2_], which were deposited on carbon nanofibers [66], were used as catalyst. The catalyst demonstrated excellent long-term stability without any deactivation during 8 h of continuous flow reactor work. In addition, no oligomers were observed during the process, and the product was of high purity. Additionally, hydrogenation of 1,5-cyclooctadiene and nitrobenzene was carried out in the gas phase with SILP system using a nickel-molybdenum carrier with a palladium catalyst [67]. Due to the magnetic properties of the IL, even traces of the catalyst can be removed from the product using a magnetic field. The addition of IL-based catalyst enabled a dramatic increase in the conversion rate from 11.6% to 99% for 1,5-cyclooctadiene and from 67.2% to 97% for the hydrogenation of nitrobenzene.

**Figure 11:**
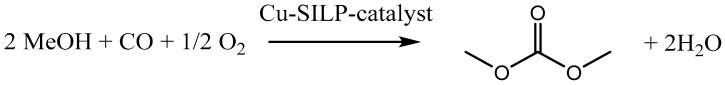
Copper based catalyst used for the synthesis of dimethyl carbonate. Reprinted with permission from Ref. [68]. Copyright 2014, Elsevier.

SILP catalyst was also designed for oxycarbonylation, with copper catalyst mixed with ILs and deposited on a polymer-based spherical activated carbon (Figure 11) [68]. The following ILs were tested: [BMIM][NTf_2_], [BMIM][OTf], 1-butyl-3-methylimidazolium acetate [BMIM][OAc], [BMIM]Cl, tetrabutylammonium chloride [Bu_4_N]Cl], N-propylpyridinium chloride [PrPyr]Cl, tetraethylammonium chloride tetrahydrate [Et_4_N]Cl · 4H_2_O, trioctylmethylammonium chloride [OMA]Cl, and trioctylmethylammonium bromide [OMA]Br whose application cased the best results among all ILs investigated in these study. High activity was maintained, even at up to 50 h of the process. The addition of a hydrophobic IL cation and a basic or halogen anion increased the catalytic activity and stability of immobilised CuBr on a solid support.

All examples described above reveal that continuous flow processes provided significant enhancement of performance in comparison with the related batch processes.

**Figure 12:**
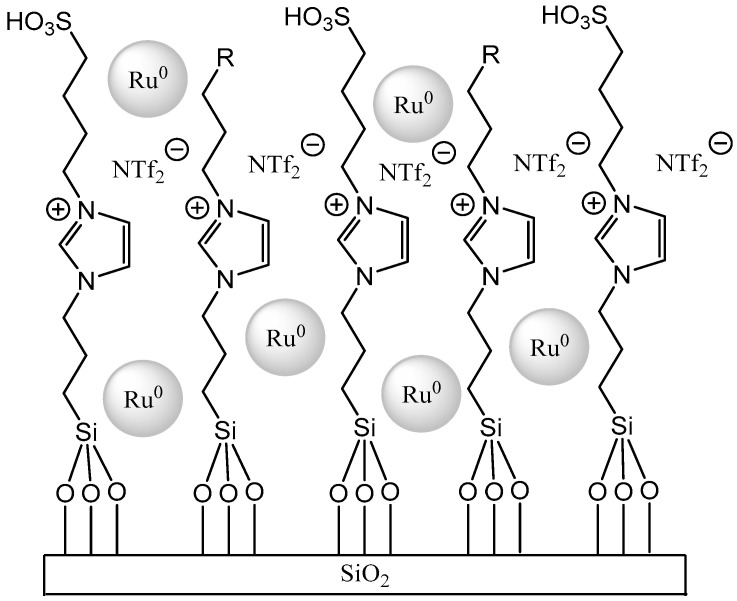
Bifunctional catalyst system based on Ru nanoparticles and an acidic SIPL (Ru NPs@SILP). Reprinted with permission from Ref. [69]. Copyright 2015, John Wiley & Sons.

**Figure 13:**
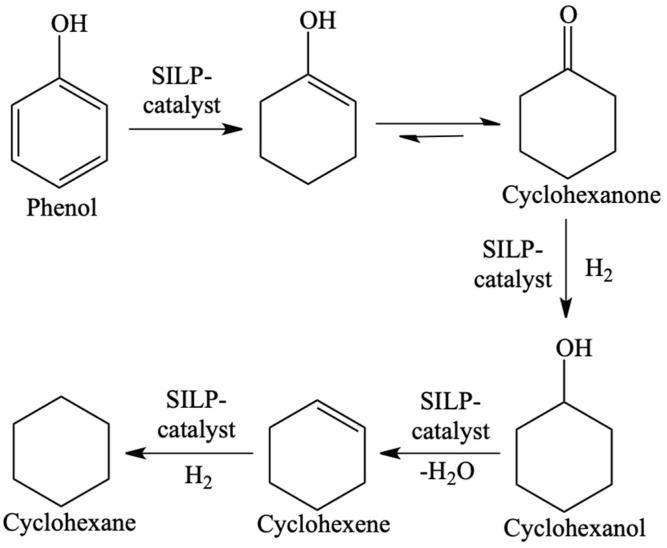
Reaction of the hydrodeoxygenation of phenol in the presence of Ru NPs@SILP as catalyst. Reprinted with permission from Ref. [69]. Copyright 2015, John Wiley & Sons.

**Figure 14:**
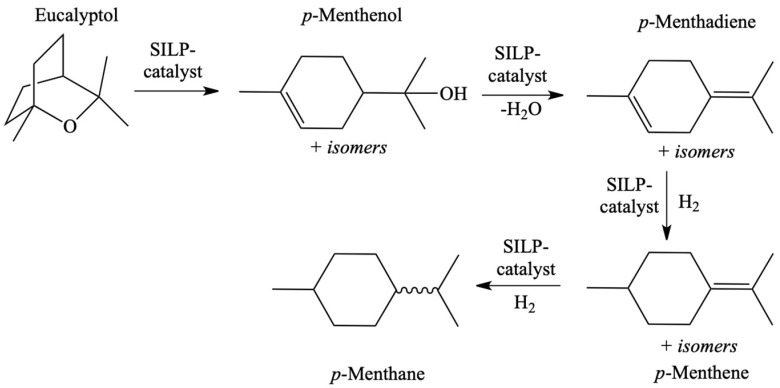
Reaction of the hydrodeoxygenation of Eucalyptol with used RuNPs@SILP as catalyst. Reprinted with permission from Ref. [70]. Copyright 2016, American Chemical Society.

The SILP catalysis and continuous flow reactors with liquid reactants were studied much more seldomly (only several publications) [69,70,71,72,73,74]. The examples included hydrodeoxygenation with ruthenium catalyst dissolved in IL deposited on silica (Figure 12) [69,70]. SILP catalysts were used in the hydrodeoxygenation of phenol (Figure 13) and hydrodeoxygenation of eucalyptol (Figure 14), allowing for the increase in the yield of products in both processes. The above example further showed that many of these catalysts are universal and one type is suitable for many chemical processes. 

**Figure 15:**
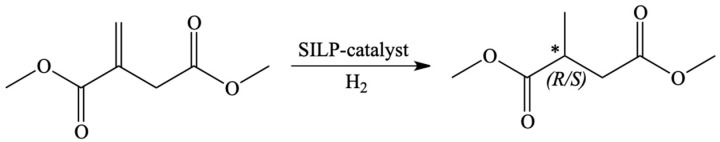
Hydrogenation of dimethylitaconate to dimethyl-2-methylsuccinate. * Chiral atom. Reprinted with permission from Ref. [71]. Copyright 2013, John Wiley & Sons.

The performance of SILP catalyst in the liquid phase was also demonstrated for enantioselective hydrogenation of dimethylitaconate to dimethyl-2-methylsuccinate (Figure 15) using a similar system as above, i.e., ruthenium catalyst dissolved in IL deposited on silica [71]. SILP catalysts were based on hydrophobic ILs [EMIM][NTf_2_] and [4-MBP][NTf_2_] (4-methyl-1-n-butylpyridinium bistrifluorosulfonamide) with the assist of supercritical CO_2_. Such a catalytic system was used for the flow reactor with productivity of 0.7 kg L^−1^h^−1^ with 100 kg of product per gram of rhodium. The use of IL allowed for increasing the activity of the catalyst and additionally extending its stability.

**Figure 16:**
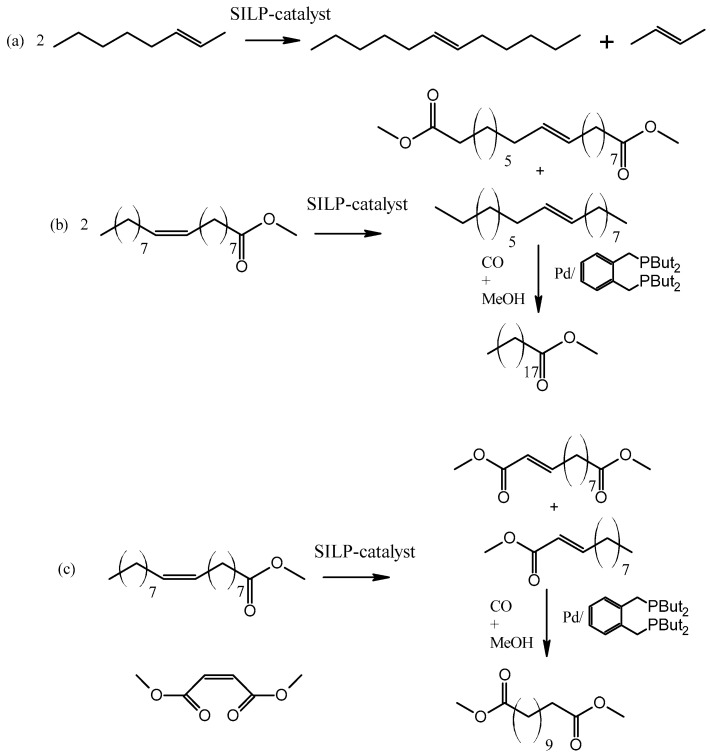
Type of the metathesis reaction (**a**) Self-metathesis of 2-octene; (**b**) self-metathesis of methyl oleate, (**c**) cross-metathesis of methyl oleate with dimethyl maleate. Reprinted with permission from Ref. [72]. Copyright 2011, Royal Society of Chemistry.

The flow system was useful for metathesis with liquid reactant as well (Figure 16). IL deposited on the surface of silica was used for Grubbs ruthenium catalyst dissolution [72]. Various ILs with [NTf_2_]^−^ anion were tested, but the most active was the one with the lipophilic [OMIM]^+^ cation. High stability of the SILP catalyst was obtained, up to 7 h with a very low content of ruthenium catalyst in the product, even below 1 ppm. IL allows for increasing the activity of the catalyst and significantly reducing the metal content in the final product.

**Figure 17:**
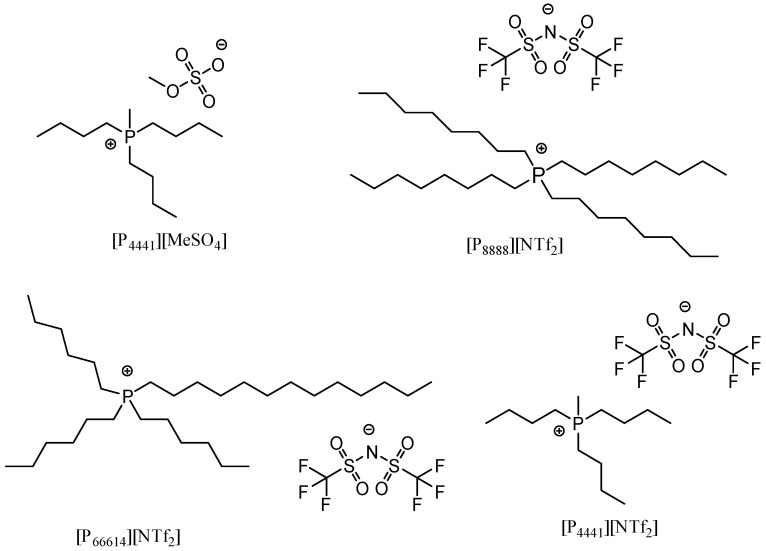
ILs used for the SILP catalysts dedicated for model hydroxylation reaction. Reprinted with permission from Ref. [73]. Copyright 2019, American Chemical Society.

**Figure 18:**
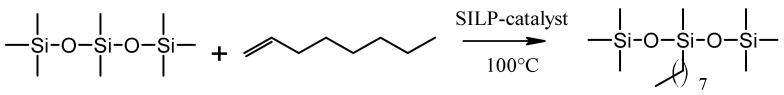
Reaction of 1,1,1,3,5,5,5-heptamethyltrisiloxane with 1-octene. Reprinted with permission from Ref. [73]. Copyright 2019, American Chemical Society.

Phosphonium ILs deposited on silica with rhodium catalyst (Figure 17) have found application in the hydroxylation reaction (Figure 18) [73]. The process assumed first the addition of a fresh portion of reagents to the catalyst, and after the reaction complication without any isolation of product, another position of reagents was added. The procedure was repeated 20 times with product accumulation. High catalytic activity was maintained in the whole experiment. This approach resulted in high TON values up to 1,796,000.

**Figure 19:**
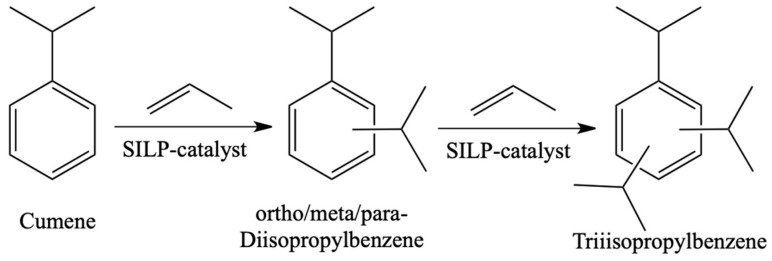
Model reaction of cumene isopropylation. Reprinted with permission from Ref. [74]. Copyright 2010, Elsevier.

The last example of the SILP system used in the liquid phase related to the alkylation of toluene and cumene in the presence of aluminium chloride deposited on silica previously modified with [EMM]Cl (Figure 19) [74]. This Lewis acidic SILP catalyst system was sensitive to moisture. Nevertheless, during the reaction carried out over 210 h, a very high stability of the system was maintained. In addition, high selectivity to the mono-alkylated product (>95%) and excellent selectivity to meta-cymene within the cymene (up to 80%) was achieved.

**Figure 20:**
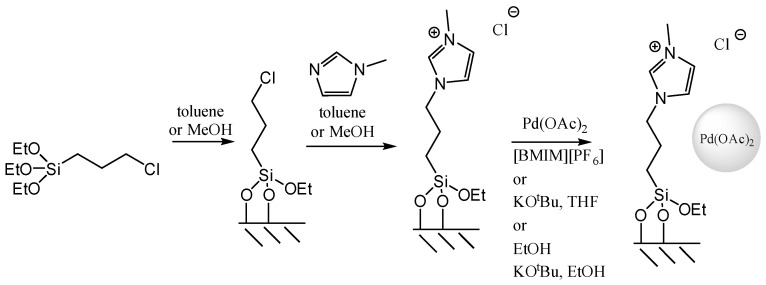
Preparation of SILP palladium catalysts. Reprinted with permission from Ref. [75]. Copyright 2014, Elsevier.

**Figure 21:**
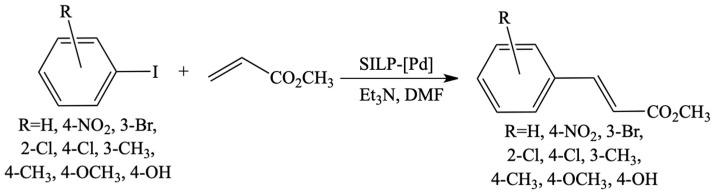
Heck reaction of methyl acrylate with aryl iodides. Reprinted with permission from Ref. [75]. Copyright 2014, Elsevier.

Another group of applications concerned SILP where IL was anchored to the carrier via covalent bond, e.g., to the silica with immobilised palladium acetate as an active phase (Figure 20) dedicated for the Heck reaction (Figure 21) [75]. The system remained active, even for 13.5 h. In addition, the amount of palladium leaching was negligible and did not exceed 0.46%.

**Figure 22:**
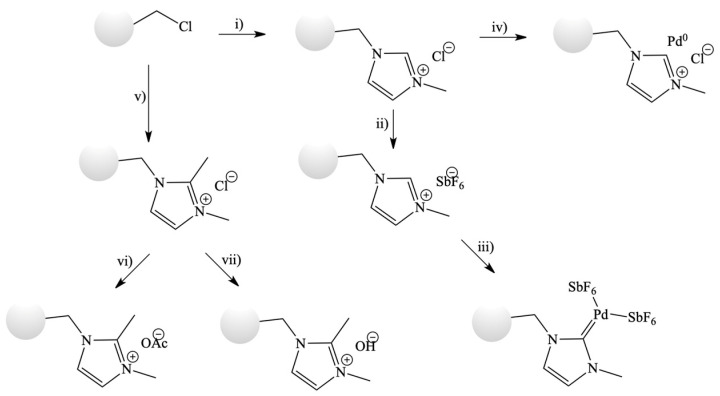
Immobilization of IL via chemical bound on the Merrifield resin. (**i**) N-methylimidazole, 80 °C; (**ii**) NaSbF_6_, MeOH; (**iii**) Pd(OAc)_2_, Na_2_CO_3_, MeOH/DMF; (**iv**) (a) Pd(OAc)_2_, HCl, THF/MeOH, (b) NaBH_4_; (**v**) 1,2-dimethylimidazole, DMF, 80 °C; (**vi**) NaOAc (aquoeus); (**vii**) NaOH (aqueous). Reprinted with permission from Ref. [76]. Copyright 2010, John Wiley & Sons.

Additionally, Merrifield resin modified with IL and palladium (Figure 22) [76] was developed for the Heck reaction. The resulting SILP catalyst was stable only for 24 h of reaction, but the degree of leaching of palladium was much lower, less than 2 ppm. Cyanosilylation [77] and Henry reaction [78] we also carried out in the water phase. Once again, ILs were bound to the Merrifield resin, but this time, ILs were neutral or alkaline. In both cases, the catalyst was highly active. Additionally, for cyanosilylation, the catalytic solvent-free system showed a very high stability for 50 h without generation of by-products.

Moreover, continuous flow synthesis with SILP catalysts was tested in biocatalysis using supports, such as silica [41], zeolite [79,80], Merrifield resin [81,82] and monolith [83]. In these cases, ionic liquids act like solvents and, more valuably, enzyme stabilisers. Ionic liquids create a protective coating surrounding protein that stabilises its folded three-dimensional structure via ionic forces, hydrogen bonds, van der Waals and hydrophobic interactions [53,84,85]. Biocatalysis is beyond the scope of this work and was the subject of other review [83,86,87]. 

## 3. Summary and Future Outlook

The use of supported ionic liquid phase (SILP) systems in chemical processes offer several advantages over traditional homogeneous catalysis, which were presented in the results obtained in the flow reactor systems. SILP systems involve the use of a solid support to hold the catalyst, making it easier to recover and reuse. This results in reduced solvent usage, improved selectivity, increased stability, and increased control over reaction conditions. These benefits, combined with the ability to achieve higher yields and greater purity of the final product, make SILP systems a highly attractive alternative to traditional homogeneous catalysis. The continued development of new and improved SILP systems holds great promise for the future of chemical production and processing, offering a sustainable and efficient alternative to traditional methods.

These benefits make SILP systems an attractive alternative to traditional methods in the field of chemical production and processing. The literature suggests that the use of SILP systems in flow reactors has the potential to greatly improve the efficiency, purity, and cost-effectiveness of chemical processes, and reduce their destructive impact on the environment.

The use of SILP systems in various reactions proved to have a positive impact in multiple ways. For example, in hydroformylation reactions, modifying the surface of silica with an ionic liquid (IL) enhanced the solubility of reactants in the IL layer, resulting in an increased conversion of reactants and improved selectivity for obtaining linear products. However, the use of SILP catalysts in the carbonylation of methanol enabled shortened reaction time and reduced the size of reactor in the described technology. In another example, using a combination of copper-based catalyst and SILP system for the synthesis of dimethyl carbonate maintained high activity, even for up to 50 h of the process. In the cited examples from the literature, the main advantages of using the SILP catalyst system include high stability of the catalyst, an increase in reaction rate, and a higher purity of the final product. As a result, the economics of the process were greatly improved by reducing operating costs or increasing reactants conversion while maintaining high selectivity. It is worth mentioning that commonly-used SILP systems based on heavy metal catalysts ensured high stability, significantly prevents its leaching and improved product purity (free of metal residue) and environmental impact.

The development of supported ionic liquid phase (SILP) techniques in flow systems holds great promise for the future of chemical production and processing. For the efficient and scalable operation of a wide range of chemical processes, such as syntheses and separations, SILP systems in flow reactors have the potential to extremely improve the efficiency, purity, and cost-effectiveness of chemical production. Additionally, the use of SILP systems can help to reduce the environmental impact of chemical processes, and improve the safety of chemical production. The study of SILP systems in flow reactors may also lead to the discovery and development of new chemical reactions, and a deeper understanding of catalytic processes. The continued development of SILP techniques in flow systems is therefore a promising and rapidly evolving area of research, with the potential to bring significant benefits to a wide range of industries.

## 4. Conclusions

To summarise, it is described in this work advantages of SILP materials and continuous flow synthesis, making this combined approach very attractive for future applications. These active, regioselective, and highly stable SILP catalysts were applied in fixed-bed continuous flow reactors for a wide range of chemical organic processes. However, as the field of SILP catalysis in continuous flow systems has only developed relatively recently, some limitations remain and significant progress is still to be expected, particularly for the processes carried out in liquid phase (few publications, have examples dominated by silica carrier and gas-phase systems). The development of supported ionic liquid phase (SILP) systems for use in flow reactors has undergone several important milestones, including:

Introduction of Ionic Liquids: The concept of using ionic liquids as support materials for catalysts in flow reactors was first introduced, marking a major shift in the field of catalytic science.

Development of Robust Supports: Researchers developed more robust and stable ionic liquid supports for catalysts, improving their stability and performance in flow reactors.

Improved Catalyst Performance: The use of SILP systems in flow reactors was shown to lead to improved catalyst activity and efficiency, compared to traditional heterogeneous catalytic systems.

Scale-Up of Flow Reactors: The development of larger and more sophisticated flow reactors enabled the scale-up of SILP systems for use in industrial applications.

Increased Understanding of SILP Systems: Researchers gained a deeper understanding of the fundamental mechanisms and properties of SILP systems, enabling the development of new and improved catalytic systems.

Development of New Catalytic Processes: The use of SILP systems in flow reactors enabled the development of new and innovative catalytic processes, such as continuous flow reactions and flow synthesis.

These milestones have led to the widespread adoption of SILP systems in flow reactors, and the continued development and improvement of these systems is an active area of research and innovation in the field of catalytic science [88]. 

## Data Availability

Not applicable.
